# Stereotactic body radiotherapy in combination with non-frontline PD-1 inhibitors and targeted agents in metastatic renal cell carcinoma

**DOI:** 10.1186/s13014-021-01937-9

**Published:** 2021-11-02

**Authors:** Yang Liu, Zhiling Zhang, Ruiqi Liu, Wensu Wei, Zitong Zhang, Lixin Mai, Shengjie Guo, Hui Han, Fangjian Zhou, Liru He, Pei Dong

**Affiliations:** 1 Department of Radiation Oncology, State Key Laboratory of Oncology in South China, Collaborative Innovation Center for Cancer Medicine, Sun Yat-Sen University Cancer Center, 651 Dongfeng Road East, Guangzhou, 510060 People’s Republic of China; 2 Department of Urology, State Key Laboratory of Oncology in South China, Collaborative Innovation Center for Cancer Medicine, Sun Yat-Sen University Cancer Center, 651 Dongfeng Road East, Guangzhou, 510060 People’s Republic of China

**Keywords:** Renal cell carcinoma, Metastasis, Anti-PD-1 therapy, Targeted therapy, Stereotactic body radiation therapy

## Abstract

**Background:**

Radiotherapy may work synergistically with immunotherapy and targeted agents. We aimed to assess the safety and outcomes of stereotactic body radiotherapy (SBRT) plus non-first-line programmed death-1 (PD-1) inhibitors and targeted agents (TA) in metastatic renal cell carcinoma (mRCC).

**Methods:**

We retrospectively reviewed 74 patients treated with non-first-line PD-1 inhibitors plus TA in non-first-line setting. Survival outcomes were calculated from the anti-PD-1 treatment using the Kaplan–Meier method. Univariate and multivariate analyses were performed by Cox proportional hazards models.

**Results:**

Thirty-two (43.2%) patients received anti-PD-1/TA therapy alone (anti-PD-1/TA alone group), and 42 (56.8%) received SBRT in addition (anti-PD-1/TA + SBRT group). The median duration of first-line therapy was 8.6 months. Patients in the anti-PD-1/TA + SBRT group had significantly longer overall survival (OS) (38.5 vs 15.4 months; *P* = 0.022). On multivariate analysis, oligometastasis, ECOG performance status 0–1, anti-PD-1/TA + SBRT, and duration of first-line therapy ≥ 8.6 months were predictors for OS. The addition of SBRT was associated with improved OS in patients with clear-cell type (HR 0.19; 95% CI 0.07–0.55; *P* = 0.002) and duration of first-line therapy ≥ 8.6 months (HR 0.22; 95% CI 0.06–0.88; *P* = 0.032). Grade ≥ 3 toxicities occurred in 23 patients (54.8%) in the anti-PD-1/TA + SBRT group, and in 21 patients (65.6%) in the anti-PD-1/TA alone group.

**Conclusions:**

Incorporating SBRT into anti-PD-1/TA therapy is safe and tolerable. Further investigation is needed, particularly in patients with clear-cell histology and a longer duration of response to first-line antiangiogenic therapy.

**Supplementary Information:**

The online version contains supplementary material available at 10.1186/s13014-021-01937-9.

## Background

Renal cell carcinoma (RCC) accounts for about 5% of all cancers in male and 3% in female in 2020 [1]. Approximately 30–40% of patients present as metastatic renal cell carcinoma (mRCC) [2]. The management of metastatic renal cell carcinoma has evolved dramatically due to the advent of immune checkpoint inhibitors (ICIs) over the past few years. First-line axitinib plus antibodies against programmed death-1 (PD-1) and its ligand has largely improved survival [3, 4], and dual PD-1 and cytotoxic T-lymphocyte associated protein 4 (CTLA-4) inhibition has also shown considerable antitumor activity in intermediate- and poor-risk patients [5]. Given these stunning results, ICIs have become an indispensable part of first-line treatment for patients with mRCC.

In the non-frontline setting, the results of ICIs appear to be less encouraging. In the CheckMate 025 trial, nivolumab is associated with a decreased risk of death, but the median progression-free survival (PFS) is only 4.6 months [6]. As for dual inhibition after anti-PD-1 therapy by nivolumab plus ipilimumab, the ORR is only 4–13%, with the median PFS of merely 3.7 months [7–9]. Combining vascular endothelial growth factor (VEGF) and PD-1 inhibitors might be promising, with an ORR of 38% in a phase I/II trial investigating sitravatinib plus nivolumab. However, toxicities requiring dose-reduction have been noted in 41.2% patients [10]. These data suggest the need to identify additional strategies to safely improve the efficacy of non-first-line ICIs in mRCC.

Stereotactic body radiotherapy (SBRT) is able to deliver highly conformal large radiation doses in limited fractions, making it an appealing choice for malignancies resistant to traditionally fractionated radiation. Accumulating evidence suggest that SBRT could provide durable local control (LC) of 90% at 1 year in mRCC, with low incidence of significant toxicity [11, 12]. Apart from local benefit, SBRT may stimulate a systemic immune response by inducing tumor cell death, modulating tumor cell phenotypes, and normalizing the aberrant tumor vasculature [13]. These preclinical findings have sparked interest in combining SBRT with ICIs in mRCC, but clinical data on SBRT combining with anti-PD-1 monotherapy and anti-PD-1/CTLA-4 therapy have polarized. The NIVES trail has only observed an ORR of 19% in patients receiving nivolumab plus SBRT, and the median PFS is only 4.1 months [14]. On the contrary, the RADVAX trial has found excellent tumor response after combining nivolumab/ipilimumab with SBRT [15].

Given the inconsistent findings and the lack of data on combining SBRT, immunotherapy and targeted therapy, our study aimed to assess the effect of SBRT on safety and survival outcomes in mRCC patients receiving non-first-line PD-1 inhibitors and targeted agents.

## Methods

### Patients and treatment

This study was approved by our institutional review board (IRB No. B2020-057-01), and informed consent was waived. Patients diagnosed with mRCC that were treated with PD-1 inhibitors between 2013 and 2020 were retrospectively reviewed. Eligible patients were aged ≥ 18 years who received PD-1 inhibitors in combination with targeted agents after failure of prior anti-VEGF therapies. Patients receiving first-line PD-1 inhibitors, non-first-line anti-PD-1 monotherapy, or conventionally fractionated radiotherapy were excluded.

All patients were treated with non-first-line PD-1 inhibitors combined with targeted agents (anti-PD-1/TA). PD-1 inhibitors and targeted agents were continued during SBRT, with no interruption or dose modification. SBRT was indicated in oligometastatic patients, and polymetasatic patients with symptomatic sites. However, the implementation was affected by patients’ willingness and their incomes.

For SBRT treatment planning, patients underwent 3 mm slice thickness computed tomography (CT) simulation scanning with site-specific immobilisation. Magnetic resonance imaging (MRI) with contrast was generally performed for lesions locating in brain, bone or soft tissue. Four-dimensional CT was mandatory for lesions in lungs, and was recommended for lesions in upper abdomen. For each lesion, the maximum dose that could be achieved according to their vicinity to normal tissues was prescribed. Prescription dose was required to cover no less than 90% of the target. Normal tissue dose constraints followed the UK Consensus on Normal Tissue Dose Constraints for Stereotactic Radiotherapy [16]. Treatment planning was designed using volumetric intensity modulated arc therapy techniques. SBRT was delivered either once daily or every other day. Cone beam CT was mandatory before each treatment to ensure accuracy. The median biologically effective dose (BED) was calculated with α/β = 3 using the linear-quadratic model.

### Outcomes

Patient were typically followed up every 3 months, including physical examination and imaging. CT scans were generally performed; MRI scans with contrast were recommended for patients with bone metastases. Early scans were allowed when clinical deterioration was present. Oligometastasis was defined as the presence of no more than five metastatic sites outside of brain and liver. Overall survival (OS) was defined from the initiation of anti-PD-1/TA to the last follow-up or death. PFS was calculated from the start of anti-PD-1/TA to disease progression or death. Duration of first-line treatment was measured from the start of first-line therapy. LC was defined as free from local progression at sites receiving SBRT. Treatment response of bone metastases were evaluated with The University of Texas MD Anderson Cancer Center (MDA) criteria [17], and the rest were evaluated using RECIST version 1.1. Toxicities were graded according to Common Terminology Criteria for Adverse Events (CTCAE version 4.0).

### Statistical analysis

Continuous variables were compared by Mann–Whitney tests, and categorical data were compared using the chi-squared test or Fisher’s exact test. Survival outcomes were estimated by Kaplan–Meier method, and the log-rank test was used to compare the survival curves. The hazard ratios (HRs) and associated 95% confidence intervals (CIs) for OS were analysed by Cox proportional hazards model. Univariate and multivariate analyses were performed to identify prognostic factors for OS; only factors significant in the univariate analyses were incorporated in the multivariate model. A two-sided *P* value of < 0.05 was considered statistically significant. All statistical analyses were performed by SPSS version 23 (IBM Corp., Armonk, NY, USA).

## Results

### Patient and treatment characteristics

A total of 74 patients were included in the analyses. Baseline characteristics are summarized in Table 1. The median age was 53 years (range 18–83 years). Fifty patients (67.6%) had clear cell type, and 58 patients (78.4%) were classified as intermediate or high risk according to the International Metastatic Renal Cell Carcinoma Database Consortium (IMDC) criteria. Only 17 patients (23.0%) were defined as oligometastasis at the start of anti-PD-1/TA treatment. Nephrectomy was performed in 63 (85.1%) patients. All patients were treated with first-line anti-VEGF therapies. The number of patients receiving sunitinib and pazopanib as first-line therapies were 31 (41.9%) and 11 (14.9%), respectively. Sixty-three patients (85.1%) received anti-PD-1/TA as second-line treatment, and the remaining patients (14.9%) received at least two lines of prior systemic therapy. For the selection of targeted agents, 72 patients (97.3%) were treated with PD-1 inhibitors concomitantly with tyrosine kinase inhibitors (TKIs). The number of patients receiving axitinib, sunitinib, pazopanib, sorafinib, bevacizumab and everolimus together with PD-1 inhibitors were 58 (78.4%), 10 (13.5%), 3 (4.1%), 1 (1.4%), 1 (1.4%) and 1 (1.4%), respectively. Pembrolizumab, nivolumab, toripalimab, sintilimab and camrelizumab were used in 35 (47.3%), 16 (21.6%), 8 (10.8%), 13 (17.6%), 2 (2.7%) patients, respectively.

**Table 1:** Baseline characteristics (N = 74)

Characteristics	Overall	anti-PD-1/TA alone (N = 32)	anti-PD-1/TA + SBRT (N = 42)	*P*
	No. of patients (%)			
Age, median (range)	53 (18–83)	53 (18–83)	53 (24–75)	0.571
Sex				0.732
Male	54 (73.0)	24 (75.0)	30 (71.4)	
Female	20 (27.0)	8 (25.0)	12 (28.6)	
Histology				0.755
Clear cell	50 (67.6)	21 (65.6)	29 (69.0)	
Non-clear cell	24 (32.4)	11 (34.4)	13 (31.0)	
ECOG				0.253
0–1	38 (51.4)	14 (43.8)	24 (57.1)	
>1	36 (48.6)	18 (56.3)	18 (42.9)	
IMDC risk group				0.461
Favorable	16 (21.6)	5 (15.6)	11 (26.2)	
Intermediate	43 (58.1)	21 (65.6)	22 (52.4)	
Poor	15 (20.3)	6 (18.8)	9 (21.4)	
Brain metastasis	2 (2.7)	1 (3.1)	1 (2.4)	1.000
Bone metastasis	18 (24.3)	4 (12.5)	14 (33.3)	0.039
Liver metastasis	9 (12.2)	6 (18.8)	3 (7.1)	0.248
Synchronous metastasis	34 (45.9)	14 (43.8)	20 (47.6)	0.741
Oligometastasis	17 (23.0)	5 (15.6)	12 (28.6)	0.190
Nephrectomy	63 (85.1)	27 (84.4)	36 (85.7)	1.000
No. of prior therapies				1.000
1	63 (85.1)	27 (84.4)	36 (85.7)	
>1	11 (14.9)	5 (15.6)	6 (14.3)	

Forty-two patients (56.8%) received SBRT (anti-PD-1/TA + SBRT group), while 32 patients (43.2%) were treated with anti-PD-1/TA alone (anti-PD-1/TA alone group). The median time from the initiation of anti-PD-1/TA to SBRT was 1.6 months. Patients were generally similar with respect to age, sex, histological type, IMDC risk group, tumor burden, intervention of primary site, and prior systemic treatment. However, higher rates of bone metastases were observed in patients receiving SBRT (Table 1).

### Response to SBRT

A total of 71 sites received SBRT. The median number of irradiated sites per patient was one. The number of patients receiving SBRT to 1, 2, 4, 5 and 6 sites were 26 (36.6%), 11 (15.5%), 3 (4.2%), 1 (1.4%) and 1(1.4%), respectively. Among patients with oligometastasis, 4 (33.3%) receive SBRT to all metastatic sites. Forty-four (62.0%) sites were located in the bones, and 6 (8.5%) sites were located in the lungs. The most frequently prescribed dose was 30–45 Gy in 5 fractions, accounting for 73.2% cases (Additional file 1: Table S1). The median BED was 146.7 Gy (range, 65.6 Gy–237.5 Gy). The rate of complete response, partial response, stable disease, and progressive disease after SBRT were 15 (21.1%), 36 (50.7%), 18 (25.4%), and 2 (2.8%). The ORR of irradiated sites was 71.8%. Two sites developed in-field progression 7 months and 24 months after SBRT, locating in adrenal gland and cervical vertebra, respectively. The 1-year LC rate was 98.2%.

### Survival and prognostic factors

**Fig. 1:**
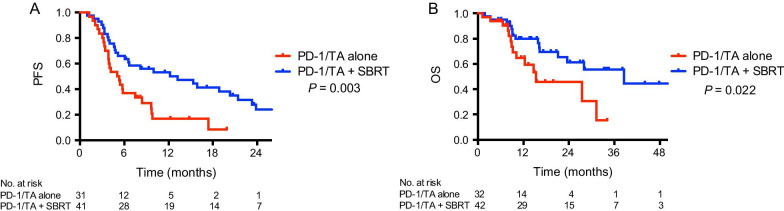
**a** Progression-free survival (N = 72) and **b** overall survival (N = 74) of patients in the anti-PD-1/TA + SBRT group and the anti-PD-1/TA alone group

After a median follow-up of 13.7 months (range 1.2–53.6 months), 29 patients (39.2%) died, and 4 patients (5.4%) were lost to follow-up. Two patients (2.7%) ceased anti-PD-1/TA therapy for intolerable side effects after 2 courses of PD-1 inhibitors. The median duration of first-line therapy was 8.6 months. For the entire cohort, the median OS was 28.0 months. In the patients treated regularly with anti-PD-1/TA, the median PFS was 6.5 months. The patients in the anti-PD-1/TA + SBRT group had longer PFS (13.2 vs 5.0 months; *P* = 0.003) and OS (38.5 vs 15.4 months; *P* = 0.022) (Fig. 1). Patients with a median duration of first-line therapy ≥ 8.6 months had significantly longer OS (38.5 vs 16.2 months; *P* = 0.041), but no significant difference was found for PFS (8.2. 0 vs 5.4 months; *P* = 0.198). No significant difference of OS was found in patients receiving different doses per fraction (not reached vs 38.5 months; *P* = 0.775) and BED (not reached vs 38.6 months; *P* = 0.864) (Additional file 1: Figure S1).

Oligometastasis at the start of anti-PD-1/TA inhibitors, ECOG performance status 0–1, anti-PD-1/TA + SBRT, and duration of first-line therapy ≥ 8.6 months were associated with superior OS on univariate analysis (Table 2). Age, sex, histology, IMDC risk group, synchronous metastasis, number of prior therapies and nephrectomy did not predict for OS. On multivariate analysis, oligometastasis, ECOG performance status 0–1, anti-PD-1/TA + SBRT, and duration of first-line therapy ≥ 8.6 months remained significant predictors for OS. The anti-PD-1/TA + SBRT treatment was associated with a 57% decreased risk of death (HR 0.43; 95% CI 0.19–0.98; *P* = 0.044) (Table 2).

**Table 2:** Prognostic factors for OS (N = 74)

Variables	Univariate analysis		Multivariate analysis	
	HR (95% CI)	*P*	HR (95% CI)	*P*
ECOG				
0–1 versus > 1	0.33 (0.15, 0.71)	0.005	0.34 (0.15, 0.78)	0.011
Oligometastasis				
Yes versus no	0.22 (0.07, 0.74)	0.015	0.28 (0.08, 0.97)	0.044
Treatment				
anti-PD-1/TA + SBRT vs anti-PD-1/TA alone	0.42 (0.20, 0.90)	0.026	0.43 (0.19, 0.98)	0.044
Duration of first-line therapy				
≥8.6 m versus 8.6 m	0.46 (0.22, 0.99)	0.046	0.42 (0.19, 0.91)	0.027

**Fig. 2:**
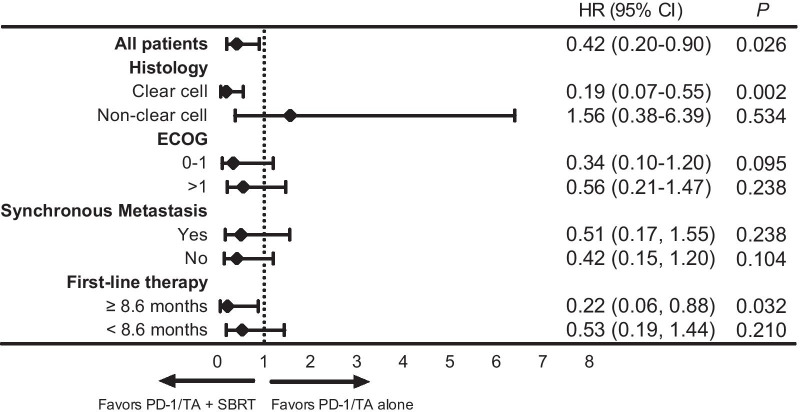
Forest plot of the association of treatment strategies and overall survival by subgroups

In order to identify potential candidates for SBRT, we analyzed the association between anti-PD-1/TA + SBRT and OS by subgroups (Fig. 2). In the subgroup of patients with clear-cell type, anti-PD-1/TA + SBRT was associated with significant improvement in OS (HR 0.19; 95% CI 0.07–0.55; *P* = 0.002). However, no improvement was found after adding SBRT to patients with non-clear-cell histology (HR 1.56; 95% CI 0.38–6.39; *P* = 0.534). In patients with duration of first-line therapy ≥ 8.6 m months, survival advantage was also observed after adding SBRT to anti-PD-1/TA inhibitors (HR 0.22; 95% CI 0.06–0.88; *P* = 0.032).

### Safety

Table 3 summarizes the adverse events (AEs) of grade 3 or higher during anti-PD-1/TA treatment. No grade 5 toxicity was reported. AEs of grade 3 or 4 occurred in 23 patients (54.8%) in the anti-PD-1/TA + SBRT group, and in 21 patients (65.6%) in the anti-PD-1/TA alone group. Fourteen immunotherapy-related AEs of any grade were reported in 13 patients (17.6%). Half of the immunotherapy-related events occurred in the anti-PD-1/TA + SBRT group. Three grade 4 AEs were observed, including one gastric perforation, one immunotherapy-related pneumonitis, and one immunotherapy-related maculopapular rash. The grade 4 immunotherapy-related pneumonitis was observed in a patient receiving intracranial SBRT in the anti-PD-1/TA + SBRT group. All the other grade 4 AEs occurred in the anti-PD-1/TA alone group. SBRT was generally well tolerated.

**Table 3:** Adverse events of grade 3 or higher during anti-PD-1/TA treatment

Event	anti-PD-1/TA alone (N = 32)	anti-PD-1/TA + SBRT (N = 42)
	No. of patients (%)	
Any adverse event of grade 3 or 4	21 (65.6)	23 (54.8)
Hypertension	4 (12.5)	5 (11.9)
Fatigue	4 (12.5)	7 (16.7)
Proteinuria	5 (15.6)	1 (2.4)
Diarrhea	3 (9.4)	4 (9.5)
Vomiting	1 (3.1)	1 (2.4)
Colonic obstruction	0 (0)	1 (2.4)
Gastric perforation	1 (3.1)	0 (0)
Hoarseness	1 (3.1)	0 (0)
Hypothyroidism	1 (3.1)	0 (0)
Creatinine increased	2 (4.8)	1 (2.4)
Neutrophil count decreased	1 (3.1)	1 (2.4)
Anemia	3 (9.4)	6 (14.3)
Palmar–plantar erythrodysesthesia syndrome	3 (9.4)	2 (4.8)
Pneumonitis	2 (4.8)	1 (2.4)
Maculopapular rash	1 (3.1)	2 (4.8)

## Discussion

Although considerable interest is directed toward combining ICIs and SBRT for patients with mRCC, evidence are still investigational at present. In this retrospective study, we found that the combination of PD-1 inhibitors, targeted agents and SBRT was tolerable in patients with mRCC. Oligometastasis, longer duration of response to first-line therapy, and the application of SBRT were significant predictors for better OS. SBRT was associated with improved survival in patients with clear-cell type and first-line therapy ≥ 8.6 months. To our knowledge, these data represent the first clinical study on the safety and efficacy of combining SBRT with non-first-line PD-1 inhibitors and targeted agents.

ICIs have led to a paradigm shift in mRCC. The potential immunological “booster” effect of SBRT has made it an appealing complement to ICIs. In mRCC, combining SBRT with PD-1 inhibitors or dual checkpoint blockade has been proved to be feasible with acceptable safety profile [14, 15]. Although anti-PD-1/TA inhibitors have shown remarkable anti-tumor effect, combining SBRT with this regimen is less frequent, possibly due to the common concerns on side effects related to concurrent use of SBRT and targeted agents. Nevertheless, prospective studies demonstrated that concurrent SBRT and targeted agents neither increase toxicities inside the irradiated field, nor increase the toxicities of anti-VEGF agents [18, 19]. In our study, the incidence of grade 3 or higher AEs was similar between the anti-PD-1/TA + SBRT group and the anti-PD-1/TA alone group (54.8% vs 65.6%), supporting the safety of adding SBRT to anti-PD-1/TA inhibitors. In respect of enhancing the activity of ICIs, our study found that patients in the anti-PD-1/TA + SBRT group had better survival outcomes compared with those in the anti-PD-1/TA alone group. Only two trials have published the data of combining SBRT and ICIs in mRCC at present. In the RADVAX trial, the median PFS reached 8.2 months with SBRT incorporated into the nivolumab/ipilimumab treatment [15]. However, the NIVES trial only reported a median PFS of 4.1 months after adding SBRT to nivolumab monotherapy [14]. These conflicting results may be due to the inconsistent lines, regimens and timing of ICIs between these two trials. In the studies investigating non-first-line ICIs plus targeted agents, the median PFS was 4.9–11.3 months [10, 20, 21] while the median PFS of our patients was 13.2 months in the anti-PD-1/TA + SBRT group. We believe that SBRT may enhance antitumor activity of ICIs and targeted agents, but the question remains unanswered and requires further investigation.

Duration of first-line treatment was previously found to be both a predictive and a prognostic factor in patients receiving subsequent axitinib treatment. In the post-hoc analysis of the AXIS trial, short duration of prior cytokines was associated with inferior PFS (HR 1.97; 95% CI 1.27–3.06; *P* = 0.002) and OS (HR 1.98; 95% CI 1.12–3.53; *P* = 0.017) in the second-line axitinib arm [22]. In other retrospective studies, prior long response to first-line TKIs was associated with better treatment response and longer PFS, particularly in patients treated with second-line TKIs [23]. In the case of ICIs, Auvray et al. reported that patients with a long first-line duration of response (≥ 6 months) to the dual immune checkpoint blockade had significantly longer PFS in second line [24]. Consistent with previous findings, we found that duration of response to first-line anti-VEGF therapy was an independent predictor for OS in patients treated with non-frontline anti-PD-1/TA inhibitors. What’s more, our study observed that patients with prolonged first-line PFS might benefit from the addition of SBRT to anti-PD-1/TA inhibitors. We speculate that tumors with longer control by first-line therapy might have less capacity for progression inherently. Previous contradictory findings in the impact of SBRT on patients receiving ICIs highlights the need to identify proper candidates. Patients with less aggressive tumor behavior as reflected by the duration of response to first-line therapy might be underlying candidates for local therapy.

It is noteworthy that in our study, SBRT was associated with survival improvement in the subgroup of patients with clear-cell type, but failed to demonstrate any survival benefit in patients with non-clear-cell renal cell carcinoma (nccRCC). Although the treatment strategy for nccRCC mirrors that of clear-cell type, response rates remain low. Treatment with traditional anti-VEGF agents like sunitinib could only yield an ORR of 5–17% in nccRCC [25–27], which has been slightly improved with the introduction of ICIs. In Keynote 427, the ORR of first-line pembrolizumab monotherapy was 26%, and the 1y-PFS was 25% [28]. These data could partially explain the disappointing ORR and PFS in the NIVES trial, which included 14% of nccRCC patients [14]. Effective systemic therapy is crucial in the management of mRCC, and local therapy directed at a few metastatic lesions could hardly reverse the systemic progression. Thus, to make the most advantage of SBRT, it should be performed when systemic control is achievable or foreseeable with systemic therapies.

Our study has several limitations. First, as this is a retrospective study, selection bias may exist. SBRT was ordered at the discretion of the multidisciplinary team, and patients receiving SBRT might be those who were most suitable for the treatment. Second, it is uncertain whether the various types of first-line anti-VEGF therapies affected the efficacy of subsequent anti-PD-1/TA treatment. Third, patients were treated with different lines and inconsistent types of anti-PD-1/TA treatment.

## Conclusions

Our study demonstrates that the combination of anti-PD-1/TA treatment and SBRT has an acceptable safety profile in patients with mRCC. Whether SBRT confer survival benefit in patients managed by anti-PD-1/TA treatment requires further investigation, especially in patients with clear-cell histology and a relatively long duration of response to first-line therapy.

## Supplementary Information

**Additional file 1.** **Table S1.** Dose and fraction regimens of irradiated sites (N = 71). **Figure S1.** Comparison of overall survival between different radio therapy plans.

## Data Availability

The associated data will be deposited in a data repository.

## Abbreviations

mRCC: Metastatic renal cell carcinoma
PD-1: Programmed death-1
SBRT: Stereotactic body radi
ICI: Immune checkpoint inhibitor
VEGF: Vascular endothelial growth factor
TA: Targeted agent
ORR: Objective response rate
BED: Biologically effective dose
nccRCC: Non-clear cell renal cell carcinoma
AE: Adverse events
