# Supplementary material for: Stereotactic body radiotherapy in combination with non-frontline PD-1 inhibitors and targeted agents in metastatic renal cell carcinoma
Source: Radiat Oncol. 2021 Nov 2;16:211. doi: 10.1186/s13014-021-01937-9 (PMC8561986; doi:10.1186/s13014-021-01937-9)
Supplement: Supplementary file 1 — Additional file 1. Table S1. Dose and fraction regimens of irradiated sites (N = 71). Figure S1. Comparison of overall survival between different radio therapy plans. [file 13014_2021_1937_MOESM1_ESM.docx]

**Supplementary Material**

Table S1. Dose and fraction regimens of irradiated sites (N=71)

| Total dose / fractions | N (%) | Sites |
| --- | --- | --- |
| 25-30 Gy / 5 fx | 5 (7.0) | Lmph node = 2  Soft tissue = 2  Bone = 1 |
| 31-35 Gy / 5 fx | 13 (18.3) | Bone = 10  Soft tissue = 3 |
| 36-40 Gy / 5 fx | 26 (36.6) | Bone = 21  Lung = 1  Brain = 1  Adrenal gland = 1  Soft tissue = 2 |
| 41-45 Gy / 5 fx | 13 (18.3) | Bone = 8  Adrenal gland = 2  Lung = 1  Soft tissue = 2 |
| 50 Gy/ 5 fx | 1 (1.4) | Lung = 1 |
| 36 Gy / 4 fx | 2 (2.8) | Kidney = 2 |
| Others | 11 (15.5) | Bone = 4  Lung = 3  Lymph node = 1  Brain = 2  Soft tissue = 1 |

Figure S1. Comparison of overall survival between different radiotherapy plans.
